# Comparative genomics of canine-isolated *Leishmania (Leishmania) amazonensis* from an endemic focus of visceral leishmaniasis in Governador Valadares, southeastern Brazil

**DOI:** 10.1038/srep40804

**Published:** 2017-01-16

**Authors:** Hugo O. Valdivia, Laila V. Almeida, Bruno M. Roatt, João Luís Reis-Cunha, Agnes Antônia Sampaio Pereira, Celia Gontijo, Ricardo Toshio Fujiwara, Alexandre B. Reis, Mandy J. Sanders, James A. Cotton, Daniella C. Bartholomeu

**Affiliations:** 1 Universidade Federal de Minas Gerais, Laboratório de Imunologia e Genômica de Parasitos, Instituto de Ciências Biológicas, Belo Horizonte, Brazil; 2 Centro de Investigaciones Tecnológicas, Biomédicas y Medioambientales, Peru; 3 Universidade Federal de Ouro Preto, Ouro Preto, Brazil; 4 Centro de Pesquisa René Rachou, Fiocruz, Belo Horizonte, Brazil; 5 Wellcome Trust Sanger Institute, Wellcome Genome Campus, Hinxton, Cambridge, CB10 1SA, UK

## Abstract

Leishmaniasis is a highly diverse group of diseases caused by kinetoplastid of the genus *Leishmania*. These parasites are taxonomically diverse, with human pathogenic species separated into two subgenera according to their development site inside the alimentary tract of the sand fly insect vector. The disease encompasses a variable spectrum of clinical manifestations with tegumentary or visceral symptoms. Among the causative species in Brazil, *Leishmania (Leishmania) amazonensis* is an important etiological agent of human cutaneous leishmaniasis that accounts for more than 8% of all cases in endemic regions. *L. (L.) amazonensis* is generally found in the north and northeast regions of Brazil. Here, we report the first isolation of *L. (L.) amazonensis* from dogs with clinical manifestations of visceral leishmaniasis in Governador Valadares, an endemic focus in the southeastern Brazilian State of Minas Gerais where *L. (L.) infantum* is also endemic. These isolates were characterized in terms of SNPs, chromosome and gene copy number variations, confirming that they are closely related to a previously sequenced isolate obtained in 1973 from the typical Northern range of this species. The results presented in this article will increase our knowledge of *L. (L.) amazonensis-*specific adaptations to infection, parasite survival and the transmission of this Amazonian species in a new endemic area of Brazil.

Leishmaniasis encompasses a group of diverse clinical diseases caused by protozoan parasites of the genus *Leishmania*. These diseases are endemic in 98 countries and pose a risk to 350 million people, with 1.5 million new cases per year^1^,^2^. *Leishmania* are digenetic organisms that live one phase of their lifecycle in an insect host from the genus *Lutomzyia* in the New World or *Phlebotomus* in the Old World and the other stage inside a mammalian host. To cope with the different environments of the invertebrate and mammalian hosts, *Leishmania* parasites present two different developmental stages: a motile flagellated extracellular promastigote form that develops within the digestive tract of the insect vector and a non-motile intracellular amastigote form that infects macrophages in the vertebrate host^3^.

The *Leishmania* genus comprises up to 35 different species, of which at least 20 are pathogenic to humans. Most clinically relevant species have been classified into two distinct subgenera (*Leishmania* and *Viannia*) according to their development site inside the alimentary tract of the sand fly^4^. Species from the *Viannia* subgenus present a phase of development at the hindgut and posterior migration to the midgut, whereas species from the *Leishmania* subgenus undergo intraluminal development in the midgut and foregut^4^. Some subsequent studies have added additional levels of complexity to this original classification^5^,^6^. Nevertheless, these two subgenera largely represent monophyletic groups^7^, although hindgut development appears to be ancestral.

Leishmaniasis is known to encompass a broad spectrum of clinical manifestations, with different symptoms being primarily associated with infections with different *Leishmania* species^2^. These distinct disease features have been classified into tegumentary (TL; also known as cutaneous leishmaniasis or CL) and visceral leishmaniasis (VL).

Among the species associated to TL in Brazil, *L. (L.) amazonensis* accounts for more than 8% of all cases in the northern and northeastern regions^8^,^9^ and is considered the main etiological agent of diffuse cutaneous leishmaniasis (DCL), which is a type of TL characterized by the appearance of multiple non-ulcerative lesions^10^.

Leishmaniasis is considered a re-emergent and emergent disease with geographical expansion due to urbanization, human migration, human-driven environmental modifications and co-infection with other diseases^11^. This expansion has led to the emergence of new foci of transmission and reactivation in previously controlled settings^12^,^13^.

The municipality of Governador Valadares in the southeastern Brazilian state of Minas Gerais is an endemic area for TL and a focus of intense transmission of VL.

The history of leishmaniasis in Governador Valadares shows that this area was highly endemic for VL during the 60 s and this disease was gradually controlled by surveillance and control activities that were implemented up to the 90 s^12^. However, an interruption of control and surveillance activities since that decade resulted in the widespread of canine VL and the reappearance of human VL in 2008^12^.

Currently, VL mainly affects males and children in Governador Valadares from 0 to 9 years of age, with a case fatality rate of 16%^12^. Epidemiological records collected since 2007 to 2013 report 127 human VL cases and 30% seropositivity in domestic dogs^12^.

Sand fly surveillance studies conducted in the region have found a high sand fly diversity with more than 10 distinct species with the predominance of *Lu. intermedia, Lutzomyia cortelezzi* and *Lutzomyia longipalpis* in the sand fly population^14^.

Epidemiological studies conducted in the region have considered *L. (L.) infantum* as the sole etiological agent of disease based on the presence of *Lutzomyia longipalpis* that is the main vector of this species^12^ and the visceral symptoms of infected human patients^12^. However, identification of *Leishmania* species using molecular or biochemical tools has not been conducted in this area.

In this article, we present the results of a comparative genomic analysis of two *L. (L.) amazonensis* strains isolated from dogs with clinical manifestations of visceral leishmaniasis in the city of Governador Valadares. Two different genome assemblies of the same *L. (L.) amazonensis* isolate (MHOM/BR/71973/M2269) have previously been published^15^,^16^. The M2269 isolate was obtained from a human cutaneous lesion in Para state, Brazil in 1973^15^. Our data is thus, to our knowledge, the first genomic data from a canine *L. (L.) amazonensis* isolate, and the first from Southeastern Brazil.

Our study has found important differences in terms of SNPs, chromosome and gene copy number variation within *L. (L.) amazonensis* and with the closely related species *L. (L.) mexicana* that have not been explored previously. As well as expanding our knowledge of the diversity and epidemiology of *L. (L.) amazonensis*, this information will ultimately contribute to the understanding of some of the mechanisms of *L. (L.) amazonensis* infection and survival as well as provide conclusive evidence of the presence of this species in VL-endemic urban areas.

## Results

### Sample collection, serology test, genotyping and genomic sequencing

We recovered 36 *Leishmania* isolates from *in vitro* culture of lymph node aspirates from dogs with clinical manifestation of visceral leishmaniasis. The symptoms included weight loss, lymphadenopathy, conjunctivitis, keratitis, anemia, ulcers, alopecia, dermatitis, onychogryphosis (abnormal nail growth) and vasculitis (Supplementary Fig. S1). Sera from these dogs had positive results using the leishmaniasis ELISA EIE-LVC kit (Supplementary Fig. S2).

Molecular genotyping indicates that isolates S3 and S6 belonged to the *Leishmania mexicana* complex due to the matched restriction profile with *L. (L.) amazonensis*/*L. (L.) mexicana* (Supplementary Fig. S3) whereas the remaining 34 isolates were genotyped as *L. (L.) infantum*.

Genomes from S3 and S6 and three *L. (L.) infantum* isolates were sequenced on the Illumina HiSeq 2000 v3 platform at The Wellcome Trust Sanger Institute. Raw sequence data was deposited in the European Nucleotide Archive with the accession number ERP016755.

### Mapping and PCA

We mapped reads against a reference sequence assembly for *L. (L.) infantum* JPCM5^17^ and called single nucleotide polymorphism (SNP) variants and filtered by read depth, base and mapping quality as described in the methods. This approach identified 23,921 and 17,624 SNPs between JPCM5 and isolates S3 and S6, respectively. Of these, 13,474 variants were shared between both isolates. This result contrasts with the lower number of variants called in the other three *L. (L.) infantum* isolates against the JPCM5 (2,342; 2,318 and 2,149 SNPs in S1, S2 and S4, respectively).

**Figure 1:**
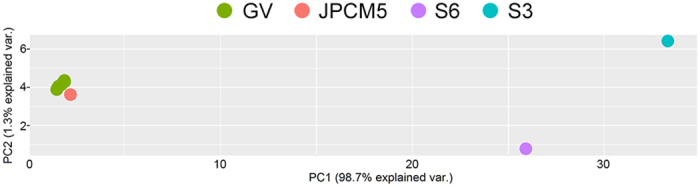
PCA of SNPs of Governador Valadares isolates: GV: three Governador Valadares *L. (L.) infantum* isolates; JPCM5: *L. (L.) infantum* JPCM5 reference strain. PCA shows that the S3 and S6 isolates to do not group with *L. (L.) infantum*.

This finding is consistent with PCA result for these isolates that shows a cluster of three isolates with the JPCM5 reference strain, while samples S3 and S6 were markedly different (Fig. 1). This result confirmed that isolates S3 and S6 were somehow unrelated to the rest of the isolates and therefore required further analysis.

Competitive mapping against the *L. (L.) mexicana* U1103 and the *L. (L.) amazonensis* M2269 reference genomes resulted in more than 78% of the reads from both the S3 and S6 isolates mapping specifically to *L. (L.) amazonensis* M2269 with a median genome coverage of 40.5 and 29.7, respectively (Supplementary Fig. S4). This result suggested that both samples had a closer relationship to *L. (L.) amazonensis* than to either *L. (L) infantum* or *L. (L.) mexicana.*

### Genome assembly

To confirm that samples S3 and S6 were indeed *L. (L.) amazonensis*, we employed a hybrid assembly approach to generate a draft genome sequence for each isolate^18^. This method resulted in 3,584 and 3,236 contigs with an N50 of 29,346 bp and 26,692 bp for isolates S3 and S6, respectively (Table 1). While still a draft, our assembly is more contiguous than either of the published *L. (L.) amazonensis* M2269 assemblies^15^,^16^. These contigs comprised more than 30.5 Mbp, which is slightly larger than the current *L. (L.) amazonensis* M2269 reference genome (version from 2013-07-25) and closer to the expected size for *Leishmania* genomes of 32 Mbp^19^. The resulting contigs were subsequently ordered into 34 pseudochromosomes assuming a similar chromosomal organization to the most closely related species available (*L. (L.) mexicana*).

### Phylogenetic inference

Comparing our assemblies for S3 and S6 against seven species, we identified 294 single-copy orthologous loci (302,742 nucleotides) for phylogenetic analysis. We identified the TVM model with invariable sites as the best model for the concatenated nucleotide dataset.

**Figure 2:**
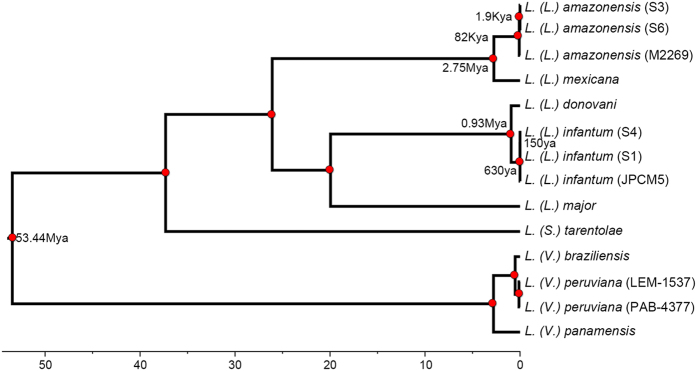
Bayesian based divergence time analysis under the relaxed clock model. Concatenated dataset 294 loci (302,742 nucleotides). The x-axis shows absolute time scale in millions of years and nodes are located at the mean divergence. This result shows that isolates S3 and S6 correspond to *L. (L.) amazonensis* and have an 82Ky divergence from the M2269 *L. (L.) amazonensis* strain. All nodes in the tree are supported with a posterior probability of one (red circles on figure).

Bayesian divergence estimation provided strong statistical support for all nodes (Fig. 2). The resulting tree supports a common origin of isolates S3 and S6 together with *L. (L.) amazonensis* M2269, clearly indicating that they belong to this species. Furthermore, we identified 15,550 and 16,178 SNPs in relation to the M2269 reference strain in S6 and S3, respectively. Of these 14,369 SNPs were shared between both isolates. This result suggests the existence of important variability within *L. (L.) amazonensis* that could be related to the distinct geographical location of these isolates and a potential ancient dispersion of this species in Brazil.

In this sense, our divergence analysis resulted in fairly similar dates to other studies previously conducted^20^,^21^ with an estimated divergence time of the *Leishmania* and *Viannia* subgenus of 53 Mya (66–40 Mya, CI 95%). Our results for *L. (L.) amazonensis* suggest that its presence in the southern regions of Brazil does not correspond to a recent expansion event but to a more ancient dispersion. We estimated that the most recent common ancestor of our two Southern *L. (L.) amazonensis* isolates and the Northern reference isolate existed around 82,000 years (120–48Kya) ago (Fig. 2). Additionally, the most recent common ancestor of S3 and S6 existed around 1,900 years ago (4.6Kya-43ya) suggesting that the *L. (L.) amazonensis* population in the vicinity of Governador Valadares could have been present for more than 2,000 years.

### Chromosome copy number variations

**Figure 3:**
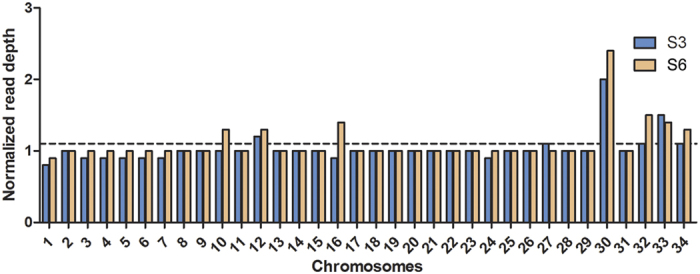
Chromosome copy number variation in *L. (L.) amazonensis* isolates S3 and S6. Columns represent the estimated haploid copy number for each chromosome. Mean genome ploidy is indicated by a dotted black line.

**Figure 4:**
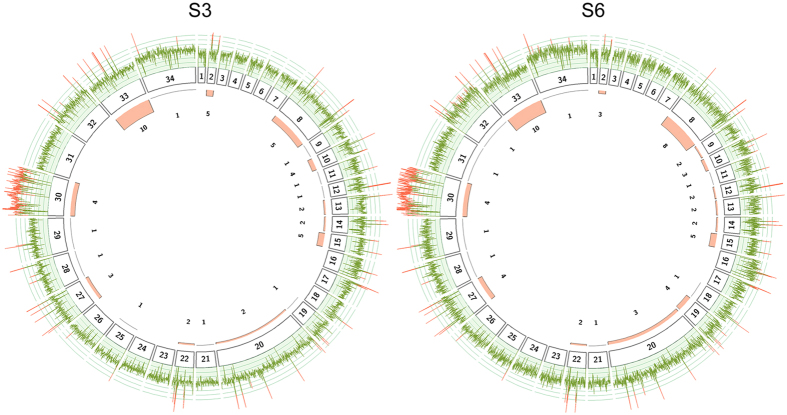
Chromosome and gene copy number variations in *L. (L.) amazonensis* isolates. The figure shows the read depth coverage on each individual chromosome (internal boxes) along with the number of expanded genes for each isolate. The mean read depth is shown as a line plot for each chromosome in green for disomic regions and on red for expanded genomic regions. For instance, read depth is evenly distributed along the entire chromosome 30 on both isolates suggesting that the whole chromosome is expanded. The internal histogram displays the total number of gene expansions identified in each chromosome indicating that most expanded genes are located on chromosome 33.

Chromosome copy numbers were estimated using the median read density of each chromosome normalized by the median read depth of the whole genome. Most chromosomes of both *L. (L.) amazonensis* isolates have a haploid copy number of one with the exception of Chr30 (Figs 3 and 4) consistent with some degrees of mosaic aneuploidy within sequenced parasite cultures^22^. This finding contrasts previous results from other studies, where a striking diversity in terms of aneuploidy was found across species^17^,^19^, different isolates from the same species^18^ and even within a single population^23^.

Chromosome 30 appears to be the only chromosome with a large increase in copy number. This chromosome is homologous to chromosome 31 of the Old World *Leishmania* and New World *Viannia* species if we assume a similar chromosomal organization to that of *L. (L.) mexicana*^13^ due to two fusion events between chromosomes 8 and 29 and between chromosomes 20 and 36^24^. In both isolates, read depth of Chr30 is homogenously distributed along the entire chromosome, supporting a complete chromosomal amplification rather than duplication of a specific chromosomal region (Fig. 4). This chromosome has been found to be polysomic in all *Leishmania* isolates sequenced to date^19^,^23^.

To confirm our estimates of chromosome ploidy for each isolate from read depth-based analyses, we examined the distribution of allele frequencies across sites for each chromosome; heterozygous sites on disomic chromosomes should have frequencies close to 0.5, while those on trisomic chromosomes will show frequencies of 1/3 or 2/3 and those on tetrasomic chromosomes can show peaks at 1/4, 1/2 and 3/4.

**Figure 5:**
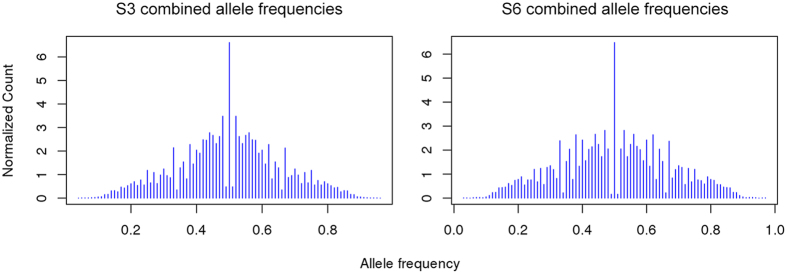
Normalized allele frequency distribution for *L. (L.) amazonensis* isolates S3 and S6. The blue lines represent normalized counts of the proportions at heterozygous positions for all chromosomes. The results support that most chromosomes are disomic in both isolates.

Allele frequency profiles for heterozygous SNPs showed a marked peak at allele frequencies close to 0.5 for most chromosomes in isolates S3 and S6 with the exception of chromosome 30 (Fig. 5, Supplementary Figs 5 and 6). The allele frequency results confirm our read depth estimates and support an overall disomic tendency for most chromosomes with the clear exception of chromosome 30 that appears to be tetrasomic in both isolates.

### Gene copy number variations

It has been suggested that gene copy number variations in *Leishmania* can affect gene expression in response to changing conditions within the host, contributing in part to the different disease tropisms that are observed in *Leishmania*^19^. Indeed, it is likely that gene dosage could play a particularly important role in regulating expression in *Leishmania* given the apparent lack of other mechanisms of transcriptional regulation in these and other kinetoplastids^25^.

The gene copy number analysis identified 53 and 62 expanded genes in S3 and S6 with 47 in common between both isolates (Fig. 4, Supplementary Tables 1 and 2). The most expanded genes included an RNA helicase, a putative pyroglutamyl peptidase I (PPI) and several hypothetical proteins, highlighting the need for characterization of trypanosomatid genes of unknown function. PPIs have been found in various organisms, but a specific biological function has not been assigned yet. These proteins hydrolyze N-terminal L-pyroglutamyl residues, which confer resistance to the modified peptides from aminopeptidase degradation and in some cases are crucial for biological activity^26^. A PPI in *Trypanosoma brucei* has been associated with protection against antimicrobial peptides, suggesting that this enzyme could be an important virulence factor^27^. However, the corresponding ortholog in *L. (L.) major* appears to be a key factor during differentiation to metacyclic promastigotes^26^. Based on this evidence, the expanded ortholog in *L. (L.) amazonensis* is also likely to act during the transition to infecting metacyclic promastigotes.

Gene ontology analysis on the expanded genes showed that this group is enriched for functions related to GTP catabolism with 11 genes totalizing 36 haploid copies (Supplementary Fig. 7). GTPase proteins are crucial in vesicle formation, motility and the union of vesicles to target compartments^28^. In *Leishmania,* GTPases play a major role during the regulation of vesicular transport in exocytic and endocytic trafficking^29^.

Another important characteristic of the *Leishmania* genomes is the presence of expanded tandem gene arrays that have been shown to vary greatly between species^19^. Our analysis found five tandem gene arrays in both *L. (L.) amazonensis* isolates (Supplementary Table 3). These tandem gene arrays include surface antigen protein 2 (PSA2), elongation factor 1 (EF-1α), ama1, HSP83 and beta tubulin.

The *Leishmania* surface antigen protein 2 (PSA-2) is a family of glycol-proteins expressed extracellularly in both parasite stages with overexpression in metacyclic promastigotes^30^. This family is involved in protecting the parasite from complement-mediated lysis^31^, and it may also be involved in host cell invasion due to the presence of leucine-rich repeats that interact with the CR-3 receptor of macrophages^32^. Our results show the presence of an expanded tandem array of five PSA2 genes in *L. (L.) amazonensis*, suggesting an important role for this virulence factor in this species (Supplementary Table 3).

*Leishmania* EF-1α is a tyrosine phosphatase-1 (SHP-1) binding protein that appears to be secreted in the phagosome. Experimental evidence shows that EF-1α targets host SHP-1 that is involved in macrophage inactivation by blocking the induction of nitric oxide synthase in response to interferon-γ^33^. Consequently, this protein reverses the phenotype of infected macrophages toward a deactivated-like phenotype that favors parasite survival^33^. An expansion of a five-EF-1α tandem array was found in our *L. (L.) amazonensis* isolates (Supplementary Table 3). This expansion, which is absent in the TL-causing *L. (L.) mexicana*, may be particularly important for the more aggressive disease phenotype associated with *L. (L.) amazonensis* that can range from DCL to VL infection.

We also found an expanded tandem gene array of AMA1 that appears to be unique in *L. (L.) amazonensis*. Although AMA1 have not been fully characterized in *Leishmania,* these genes might be involved in parasite interaction with host membrane cholesterol promoting parasite invasion^34^.

We were also able to find an expansion in a tandem gene array of three HSPs located in chromosome 32. These proteins maintain protein folding under stress conditions such as the ones inside the phagosome and participate in differentiation during the lifecycle of *Leishmania*^35^.

## Discussion

*L. (L.) amazonensis* is an important cause of tegumentary leishmaniasis in Brazil in the northern and northeastern regions of the country^8^,^9^. As part of a study aiming at characterizing *Leishmania* isolates circulating in the city of Governador Valadares, Minas Gerais state, Brazil, we have sequenced the genomes of several *Leishmania* isolates from this focus. Genome sequencing of five isolates obtained from dogs revealed the presence of *L. (L.) amazonensis* in this endemic region of tegumentary and visceral leishmaniasis^36^. The genomic analysis performed in this study of the two *L. (L.) amazonensis* isolates allowed us to explore some unique features in terms of chromosome and gene copy number variations that are unique to this species.

Our results clearly show that most *L. (L.) amazonensis* chromosomes are disomic in contrast to other analyzed *Leishmania* species where genome plasticity and mosaic aneuploidy is a more common trait^18^,^19^. Mosaic aneuploidy has been proposed as a rapid adaptive mechanism in *Leishmania* to address different conditions inside its hosts^19^,^37^. The largely disomic pattern observed in *L. (L.) amazonensis* could be the result of different selection pressures to other *Leishmania* species.

Gene copy number variations in relation to *L. (L.) mexicana* show that species-specific expansions exist despite the high similarity, especially in expanded genes and tandem arrays in proteins potentially involved in cell differentiation, cellular trafficking and parasite host interaction.

The different sets of gene expansions in *Leishmania* known as intrachromosomal amplifications appear to serve as a mechanism to modify gene dosage in the absence of transcriptional control of gene expression^19^. In *L. (L.) amazonensis*, this mechanism could be crucial for invasion and survival inside host macrophages, playing an important role for PSA2 and EF-1α, and it may also be partially responsible for the broad clinical phenotype associated with different isolates including TL, DCL and VL.

Using information retrieved from the assembled S3 and S6 genomes, we explored the presence of reported markers implicated in visceralization in these two *L. (L.) amazonensis* isolates. Unfortunately, the gene that encodes the A2 protein that is the prototype of visceralization is collapsed in both assemblies due to its large repetitive region. Nevertheless, we found the gene LinJ.15.0900 (nucleotide sugar transporter) whose ortholog in *L. (L.) donovani* have been implicated in increased parasite burden in the liver (18 fold) when expressed in *L. (L.) major*^38^. This gene, which is absent in *L. (V.) braziliensis* and a pseudogene in *L. (L). major,* could be an important promoter of visceralization. In addition, it could also has a more general role in virulence due to the fact that it also promotes footpad swelling^39^. In this sense, this finding reveals the complexity of VL that likely involves the combination of different parasite specific genetic factors as well as the host immune response^40^. It is important to emphasize that because we are analyzing only two *L. amazonensis* isolates, it is difficult to obtain robust information about determinants of the disease. This question should be addressed in a larger study with an increased number of *L. amazonensis* isolates and oriented towards a comparative perspective against a VL species, like *L. infantum.*

Governador Valadares city is a re-emergent focus of visceral leishmaniasis with a high number of human cases due to *L. (L.) infantum* and a high prevalence of infected dogs^36^. To our knowledge, this article presents the first report of *L. (L.) amazonensis* in Governador Valadares and shows a potential risk for current control efforts in the area that have been designed considering only the presence of *L. (L.) infantum*.

Importantly, both dogs infected with *L. (L.) amazonensis* presented all clinical symptoms shown by dogs infected with *L. (L.) infantum* in the same area. This information provides evidence of the severity of *L. (L) amazonensis* infection and might indicate potential involvement of *L. (L.) amazonensis* in canine VL that should be further investigated.

The isolation of *L. (L.) amazonensis* from domestic dogs indicates a possible domestic cycle of *L. (L.) amazonensis* posing an increased risk of transmission to humans and possibly showing an urbanization process of this species. This finding also underscores the lack of knowledge regarding the distribution of this species in Brazil, complementing previous isolations of *L. (V.) amazonensis* in dogs with clinical VL diagnosis in other southern Brazilian regions in the states of Sao Paulo^41^ and Minas Gerais^42^.

The finding of *L. (L.) amazonensis* in different ecological niches than the ones in the northern and northeastern regions of Brazil stresses the need to revise the current serological and molecular *Leishmania* detection tests employed in endemic visceral leishmaniasis sites. Our molecular clock analysis is consistent with previous findings of an ancient divergence between the two *Leishmania* subgenera, although the relatively recent date (53.4 mya) could support the idea that multiple trans-continental dispersal events^43^ rather than vicariance^6^ explains the current geographical distribution of these species. We also find evidence that the presence of *L. (L.) amazonensis* in southern Brazilian regions correspond to an ancient dispersion event rather than a recent introduction of this species, as our two southern isolates are estimated to have a common ancestor almost two thousand years ago, and to have diverged from a Northern *L. amazonensis* isolate over 80,000 years ago. Sequence data from additional isolates from both foci would help to shed further lights on the epidemiology of *L. (L.) amazonensis*, and exclude an alternative explanation of multiple introductions of this species to Southern Brazil.

Our results indicate that the EIE-LVC kit and possibly other diagnostic kits based on similar antigens can cross-react with *L. (L.) amazonensis*. This cross-reactivity may have resulted in an underestimation of the distribution and prevalence of *L. (L.) amazonensis* in Brazil. This important drawback underscores the need to develop better diagnostic methods capable of discriminating between *L. (L.) amazonensis* and *L. (L.) infantum* and to encourage the use of genomics into epidemiological studies. Given the different clinical presentations of human infections with *L. (L.) amazonensis* and *L. (L.) infantum*, surveillance efforts and control activities in this region should consider the presence of *L. (L.) amazonensis* and address putative vectors for this species and the risks of co-infections in human subjects.

## Methods

### Study site and sample collection

**Figure 6:**
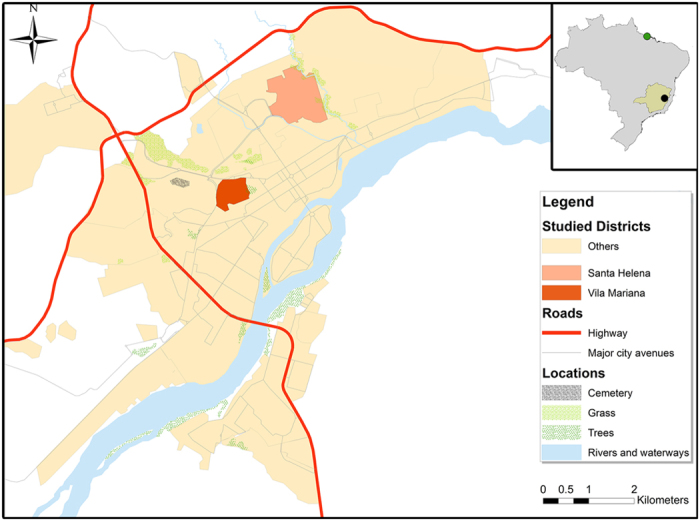
City map of Governador Valadares created using ArcGIS V.10.0. The upper right inset shows the location of the Minas Gerais State in Brazil, the city of Governador Valadares (black circle) and the isolation site of the M2269 *L. (L.) amazonensis* reference in the State of Para (green circle). On the left, we show the neighborhoods where isolates S3 and S6 were collected in Governador Valadares (Vila Mariana and Santa Helena, respectively).

Samples were taken starting in 2008 from domestic dogs with clinical VL symptoms from the endemic focus of Governador Valadares in the southeastern Brazilian State of Minas Gerais (Fig. 6). This city of approximately 280,000 inhabitants is located at the bank of the Doce River at 455 meters above sea level. The region presents a tropical sub-humid climate^44^ with a mean annual temperature of 29 °C and a mean annual precipitation of 1,059 mm. The area is endemic for TL and a reemerging focus of VL with 127 human cases of VL reported between 2007 and 2013^45^ and where more than 30% of domestic dogs are positive by serology^12^. We collected bone marrow or lymph node aspirates and serum for each dog that were subsequently used for *in vitro* culture of *Leishmania* and serology diagnosis. All experimental procedures were approved by the Committees of Ethics in Animal Experimentation of the Universidade Federal de Ouro Preto (protocol number 083/2007) and were conducted according to the guidelines set by the Brazilian Animal Experimental College (COBEA), Federal Law number 11794.

Parasites were cultivated in Schneider culture medium supplemented with 10% fetal bovine serum and 1% penicillin and streptomycin for up to three passages. Genomic DNA was extracted from ≈10^9^ promastigotes using the DNeasy Blood and Tissue Kit (Qiagen) using the manufacturers protocol.

### Serology test and molecular genotyping

Sera from dogs were tested in triplicate by ELISA using the EIE-LVC kit supplied by Biomanguinhos following the manufacturer’s standard protocol. This kit consists of soluble antigens of *L. (L.) major* and has been widely used in public health laboratories in Brazil for the diagnosis and surveillance of canine visceral leishmaniasis.

DNA from all isolates was used for genotyping using primers specific to the *hsp70* gene followed by digestion with *Hae* III restriction enzyme, as previously described^46^.

### Genome sequencing sequencing

Based on results from molecular typing we sequenced the genomes of five selected isolates.

Genomic DNA was sheared into 400–600-base pair fragments by focused ultrasonication (Covaris Adaptive Focused Acoustics technology (AFA Inc., Woburn, USA)) and standard Illumina libraries were prepared.

These libraries were used to produce 100 base pair paired-end reads on the HiSeq 2000 v3 according to the manufacturer’s standard sequencing protocol.

The *L. (L.) infantum* JPCM5^17^*, L. (L.) mexicana* U1103^19^ and *L. (L.) amazonensis* M2269^15^ reference genomes were downloaded from version 10 of the TriTrypDB database (http://tritrypdb.org/) for the mapping and genome assembly steps.

### Mapping and PCA

Initially, reads were mapped onto the *L. (L.) infantum* JPCM5 reference genome using Bowtie2^47^ followed by SNP calling with SAMtools Mpileup^48^, selecting sites with base quality scores ≥ 30, mapping quality scores ≥ 25, minimum coverage ≥ 10 reads and less than twice the median genome coverage. These filtered SNPs were later used for PCA analysis using the Caret package in R^49^.

Isolates S3 and S6 were subsequently mapped against the *L. (L.) mexicana* U1103 and *L. (L.) amazonensis* M2269 reference strains using Bowtie2^47^ and SNPs against the M2269 reference were called.

### Genome assembly and annotation

Reads from isolates S3 and S6 were filtered by quality using Trimmomatic^50^ with a minimum base quality cutoff of 30, leading and trailing base qualities of 25, a sliding window of five bases, a minimum per base average quality of 25 and a minimum read length of 65 bp.

A combined *de novo* and reference based assembly approach^18^ was employed for each sample. Briefly, for each sample we generated a *de novo* assembly using Velvet 1.2.10^51^ and a reference-based sequence with vcfutils^48^ using the *L. (L.) amazonensis* M2269 genome as a template.

*De novo* and reference based sequences were combined in ZORRO^15^, and the resulting hybrid assembly was extended and corrected with GapFiller^52^ and iCORN2^53^. Contigs were scaffolded with SSPACE^54^ and used to generate pseudochromosomes with ABACAS^55^, assuming a similar chromosome organization as in *L. (L.) mexicana*^13^.

### Phylogenetic analysis

Protein and nucleotide sequences from nine *Leishmania* species were downloaded from release v10 of the TriTrypDB database.

For each assembled genome of *L. (L.) amazonensis* (S3 and S6) and *L. (L.) infantum* (S1 and S4) a BLASTn search^56^ was performed using a cutoff of 1e^−5^ against the *L. (L.) amazonensis* M2269 and *L. (L.) infantum* JPCM5 coding sequences (CDSs), and the best match was retrieved for each gene. Nucleotide sequences were filtered out using an in house Perl script to remove pseudogenes, partial and fragmented sequences.

CDSs from all species were used as input for OrthoMCL^57^ to select single-copy genes with shared orthologs in all *Leishmania* species in the dataset. Each ortholog group was aligned using MUSCLEv3.8^58^, and poorly aligned regions were removed with trimALv1.4^59^. For all nucleotide phylogenies, jModelTest 2.1.5^60^ was used to carry out statistical selection of best-fit models using the Akaike information criterion.

Approximate likelihood ratio-tests (aLRT) were performed in 466 ortholog groups to evaluate the null hypothesis that each locus in the dataset evolved under a molecular clock^61^. It has been suggested that significance of the aLRT can be determined by halving the p-value from a chi-square test with 1 degree of freedom^62^.

The molecular clock was not rejected in 294 loci comprising 302,742 nucleotides, which were concatenated and analyzed using Bayesian divergence time estimation analysis implemented in BEAST v2.1.2^63^ using the relaxed lognormal clock model. All analyses were conducted without any topological constraints using the general time reversible substitution model with 4 gamma rate categories selected by jModelTest 2.1.5^60^. All priors were set to default values, except for the Yule’s speciation process as a tree prior and the divergence estimate calibration points.

The calibration points were provided from divergence dates already estimated between L. (*L.) donovani* and *L (L.). infantum* (0.95 Mya, SD 0.1 Mya)^21^, between *L. (L.*) major *and L. (L.) donovani* (19.6 Mya, SD 2 Mya)^20^ and between Old World and New World *L (L.). infantum* (500 ya, SD 200 years), using a normal distribution to model the prior uncertainty in these calibration dates. Times of divergence were obtained by combining estimated from 3 independent Markov Chain Monte Carlo (MCMC) runs in order to ensure convergence between the runs. Each run had a chain length of 30 million generations, with posterior samples retained every 5000 steps of each chain. All information produced by BEAST was summarized onto a single “target” tree using the TreeAnnotator module of BEAST with Burnin of 20% of the samples and tree topology was represented using Figtree.

### Allele frequency distribution

Allele frequencies for samples S3 and S6 were generated from filtered SAMtools results^48^. Briefly, for each heterozygous site we estimated the proportion of reads mapping to the alternative and reference base. Proportions were then grouped in bins from 0.01 to 1.0 and normalized by the sum of all allele frequencies for the respective chromosome. Plots of the distribution of allele frequencies were generated in R^49^.

### Chromosome and gene copy number analysis

To estimate the haploid chromosome copy number, we normalized the median read depth for each chromosome by the median read depth of the whole genome using an in house Perl script. Figures were generated in Graph Pad Prism V5 and Circus^64^.

Gene copy number variations were assessed by single-copy gene normalization. Briefly, we used OrthoMCL^57^ to select single-copy genes with orthologs in *L. (V.) braziliensis, L. (L.) mexicana, L. (L.) major, L. (L.) infantum, L. (L.) donovani* and *L. (S.) tarentolae*. The mean read depth of these genes was then used to normalize each position along the genome. Gene copy numbers were furthered normalized by the average chromosome haploid copy number calculated from the allele frequency analysis. We employed a cutoff of 1.85 to discriminate between single-copy and expanded genes.

To find enriched functions in expanded genes, we analyzed overrepresented gene ontology codes using hypergeometric distribution analysis with the Benjamini and Hochberg false discovery rate correction implemented in BINGO^65^.

## Additional Information

**How to cite this article:** Valdivia, H. O. *et al*. Comparative genomics of canine-isolated *Leishmania (Leishmania) amazonensis* from an endemic focus of visceral leishmaniasis in Governador Valadares, southeastern Brazil. *Sci. Rep.* **7**, 40804; doi: 10.1038/srep40804 (2017).

**Publisher's note:** Springer Nature remains neutral with regard to jurisdictional claims in published maps and institutional affiliations.

## Supplementary Material

Supplementary Figures

Supplementary Tables

## Figures and Tables

**Table 1:** Genome assembly results for samples S3 and S6.

Variable\Sample	Scaffolds			Contigs			
	M2269^1^	S3	S6	M2269^1^	M2269^2^	S3	S6
Number	2,627	3,293	2,545	2,944	10,305	3,584	3,236
Size	29.0 Mb	30.8 Mb	30.5 Mb	29.0 Mb	29.6 Mb	30.8 Mb	30.5 Mb
Longest	171,320	196,967	314,951	113,027	141,211	196,967	174,893
N50	22,901	32,050	33,999	19,306	6,946	29,346	26,692
Mean size	11,050	9,364	12,002	9,854	2,879	8,601	9,425

M2269^1^ is the 2013-07-25 version of the *L. (L.) amazonensis* genome published by Real *et al*.^15^. M2269^2^ is the *L. (L.) amazonensis* genome published by Tschoeke^16^, there are no scaffolds available for this assembly.
