# Supplementary material for: Comparative genomics of canine-isolated Leishmania (Leishmania) amazonensis from an endemic focus of visceral leishmaniasis in Governador Valadares, southeastern Brazil
Source: Sci Rep. 2017 Jan 16;7:40804. doi: 10.1038/srep40804 (PMC5238499; doi:10.1038/srep40804)

Comparative genomics of canine-isolated L. (Leishmania) amazonensis from an endemic focus of visceral leishmaniasis in Governador Valadares, southeastern Brazil

Hugo O. Valdivia[1,2]; Laila V. Almeida[1]; Bruno M. Roatt[3]; Agnes Antônia Sampaio Pereira[4]; Celia Gontijo[4]; Ricardo Toshio Fujiwara[1]; Alexandre B. Reis[4]; Mandy J. Sanders[5]; James A. Cotton[5]; *Daniella C. Bartholomeu[1,2]

1 Universidade Federal de Minas Gerais, Laboratório de Imunologia e Genômica de Parasitos, Instituto de Ciências Biológicas, Belo Horizonte, Brazil

2 Centro de Investigaciones Tecnológicas, Biomédicas y Medioambientales, Peru

3 Universidade Federal de Ouro Preto, Ouro Preto, Brazil

4 Centro de Pesquisa René Rachou, Fiocruz, Belo Horizonte, Brazil

5 Wellcome Trust Sanger Institute, Wellcome Genome Campus, Hinxton, Cambridge. CB10 1SA, UK

Email addresses:

HOV: hvalrod@hotmail.com

LVA: lailavalmeida@gmail.com

BMR: bmroatt@gmail.com

AASP: agnes.pereira@cpqrr.fiocruz.br

CG: gontijo@cpqrr.fiocruz.br

RTF: fujiwara@icb.ufmg.br

ABR: alexreisufop@gmail.com

MJS: mjs@sanger.ac.uk

JAC: jc17@sanger.ac.uk

DCB: daniella@icb.ufmg.br

Supplementary Figure S1: Clinical symptomatology of infected dogs. This figure describes the clinical symptoms of sampled dogs. Dogs infected with *L. (L.) amazonensis* presented similar symptoms as the ones infected with *L. (L.) infantum* . The dog infected with isolate S6 presented all the clinical symptoms shown in the figure while the one with isolate S3 presented linfadenopathy, conjunctivitis, anaemia, ulcers, alopecia dermatitis, onychogryphosis and vasculitis.

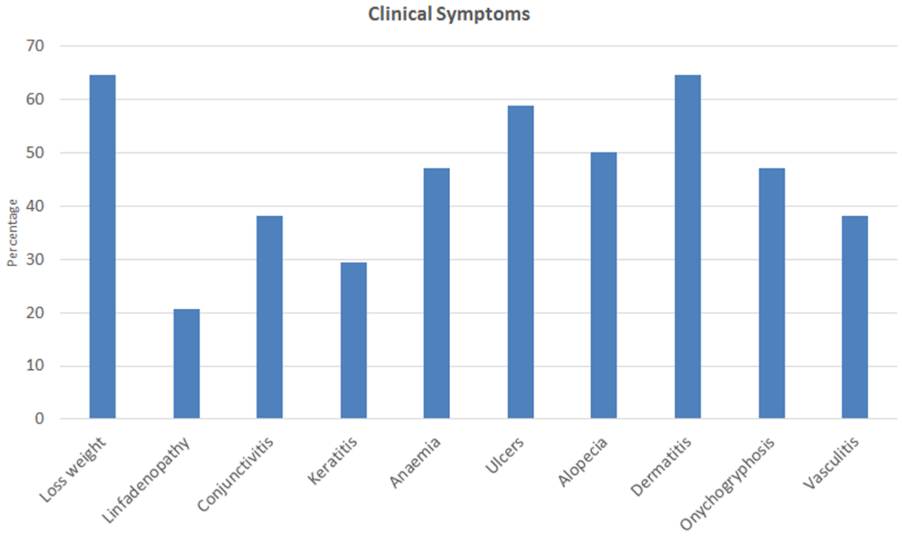

Supplementary Figure S2: ELISA EIE-LVC kit results. The colors denote samples as follows: Blue—positive and negative controls; black — *L. (L.) infantum* isolates; red — *L. (L.) amazonensis* isolates.

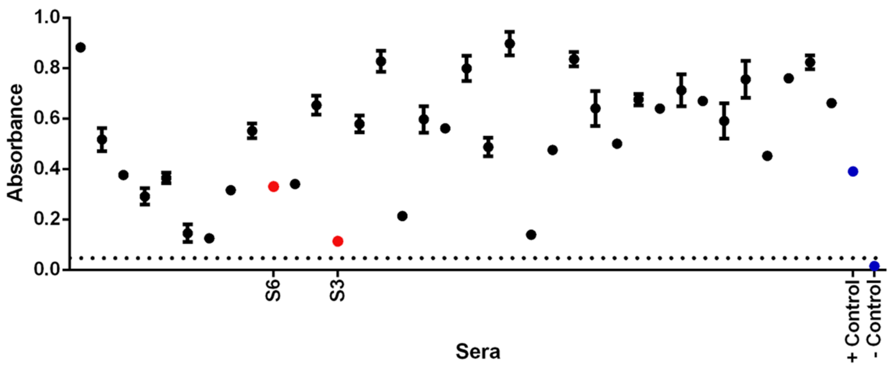

Supplementary Figure S3: Genotyping of Leishmania isolates from Governador Valadares (GV). Genomic DNA was amplified using primers specific to the hsp70 gene followed by digestion with HaeIII restriction enzyme and separation by electrophoresis in 4% agarose gel. Alphanumeric codes denote isolates collected from Governador Valadares; La/Lm – *Leishmania (L.) amazonensis* control (undistinguishable from *L. (L.)mexicana*); Lb – *L. (V.) braziliensis* control; Li – *L. (L.) infantum* control; Lg – *L. (V.) guyanensis* control

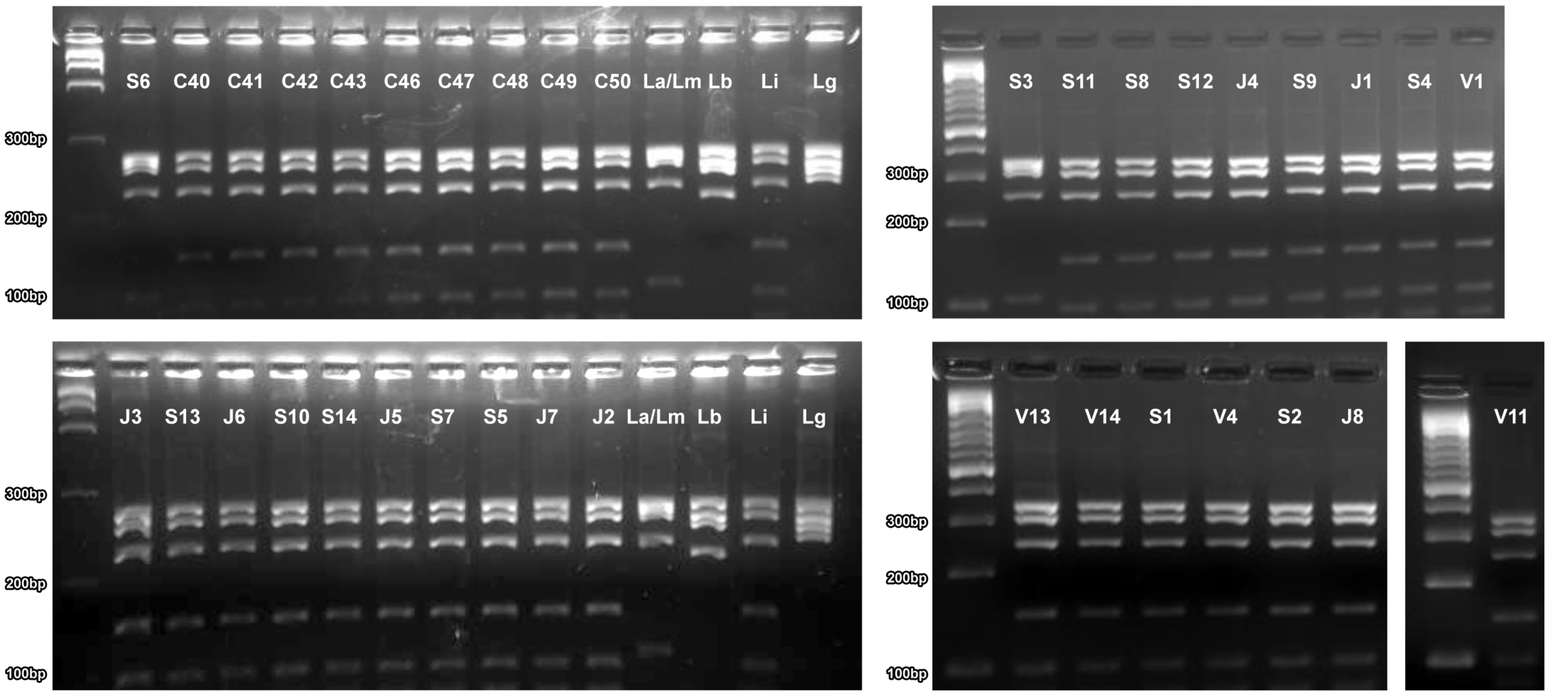

Supplementary Figure S4: Competitive mapping of *L.(L.) amazonensis* S3 and S6 reads against the reference genomes of *L.(L.) mexicana*, *L.(L.) amazonensis* and *L.(L.) infantum*. Y axis shows the number of mapped reads and the x axis denotes the species.

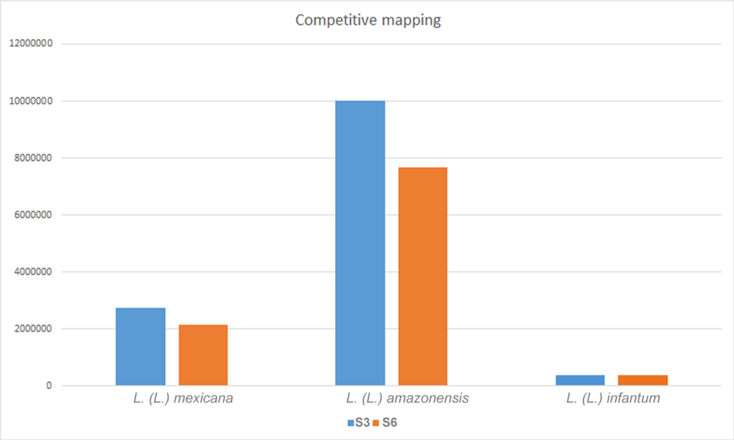

Supplementary Figure S5: Distribution of normalized allele frequency counts for the *L. (L.) amazonensis* S3 isolate. The Y axis represents the normalized counts and the X axis represents the allele frequency.

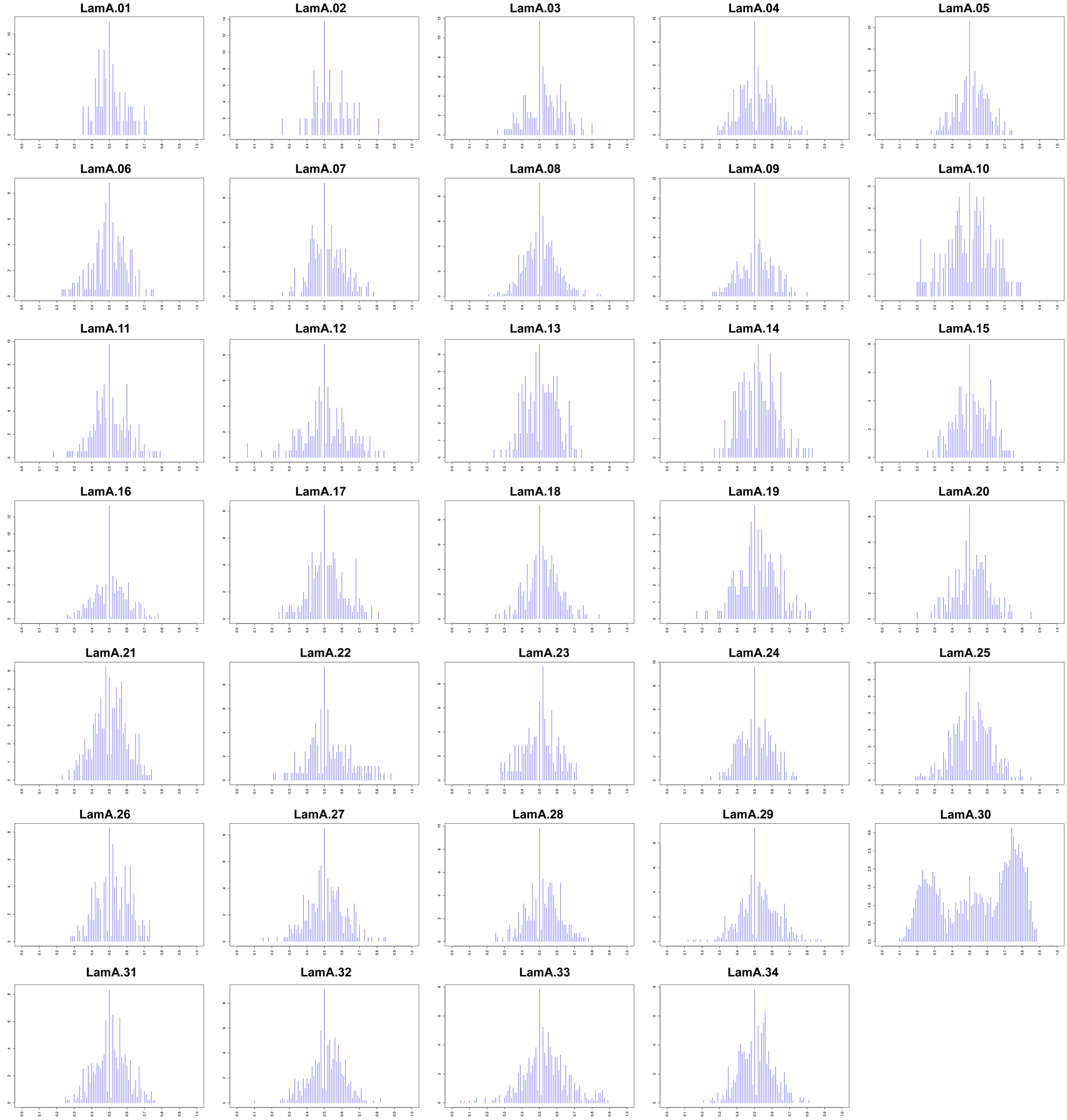

Supplementary Figure S6: Distribution of normalized allele frequency counts for the *L. (L.) amazonensis* S6 isolate. The Y axis represents the normalized counts and the X axis represents the allele frequency.

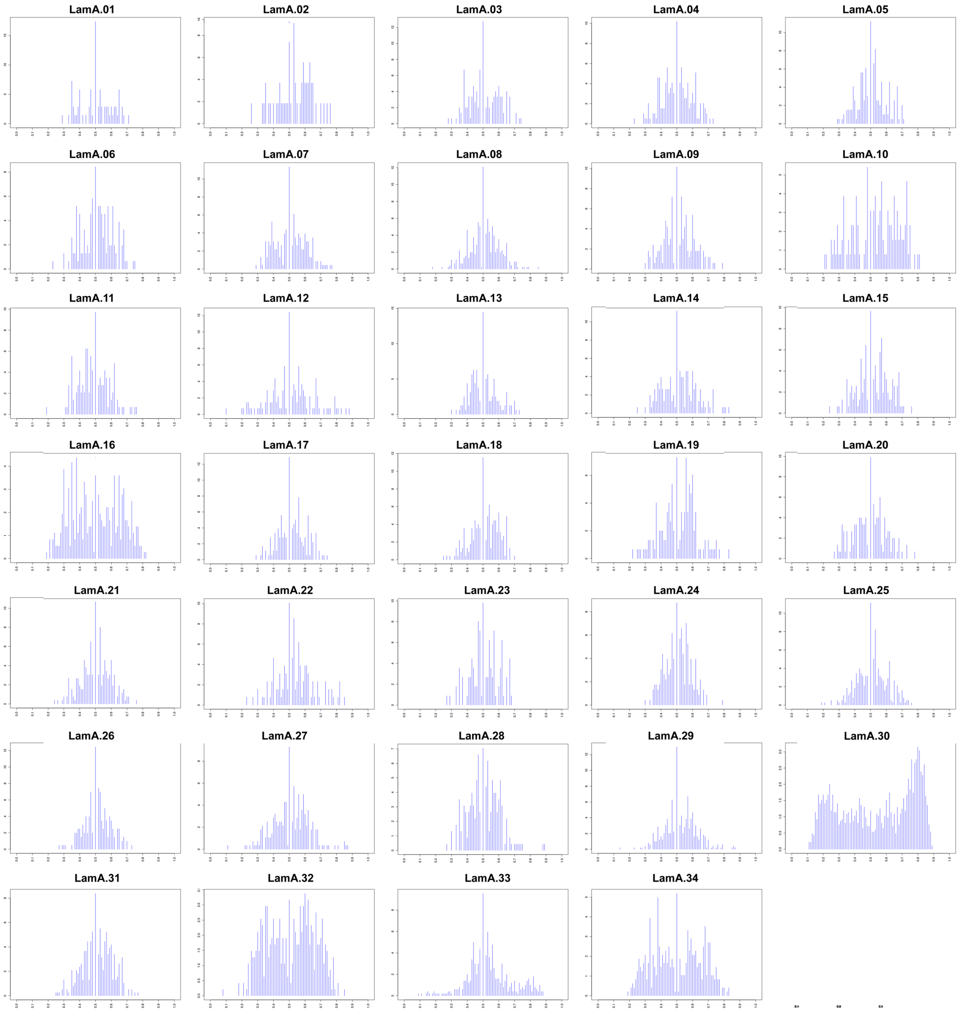

Supplementary Figure S7: Enriched statistically significant gene ontology terms in the *L. (L.) amazonensis* expansions. The y-axis show the enriched ontology term and the x-axis the number of genes associated to it.

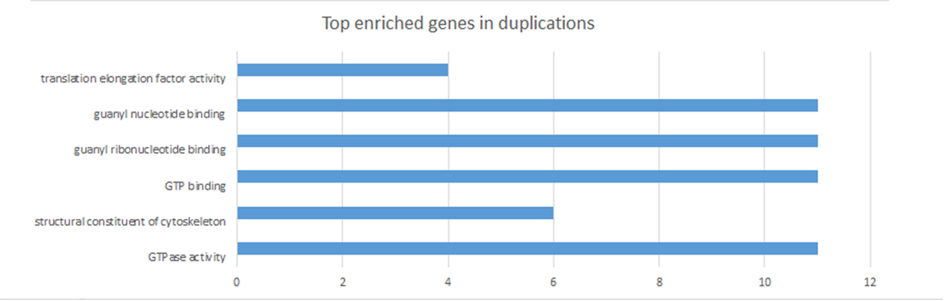

Supplement: Supplementary Figures [file srep40804-s1.pdf]
